# Supplementary material for: Enhancing the fairness of AI prediction models by Quasi-Pareto improvement among heterogeneous thyroid nodule population
Source: Nat Commun. 2024 Mar 4;15:1958. doi: 10.1038/s41467-024-44906-y (PMC10912763; doi:10.1038/s41467-024-44906-y)
Supplement: Supplementary file 1 — Supplementary Information [file 41467_2024_44906_MOESM1_ESM.pdf]

Enhancing the Fairness of AI among Heterogeneous Thyroid Nodule Population by Quasi-Pareto Improvement

Supplementary Information

Supplementary Tables

	Loss				Subgroup (Dominant & Less-prevalent)	AUROC				
	Adaptive weight in L_y	$\gamma_d L_d(\omega_y, \omega_y)$	$\gamma_{MMD} L_{MMD}(\omega_Z)$	$\gamma_{BSS} L_{BSS}(\omega_Z)$		Train	Valid	Test	Test (Dominant)	Test (Less-prevalent)
	δ	$\gamma_d = 0.2$	$\gamma_{MMD} = 1$	$\gamma_{BSS} = 0.5$						
(1)					Papillary & Follicular	0.9788	0.8486	0.8572	0.8804	0.6741
					Papillary & Medullary	0.9327	0.8363	0.8471	0.8847	0.6502
					Tertiary & Community	0.9411	0.8415	0.8489	0.8852	0.6970
(2)	√				Papillary & Follicular	0.9676	0.8344	0.8525	0.8659	0.7022
					Papillary & Medullary	0.9483	0.8470	0.8244	0.8764	0.6736
					Tertiary & Community	0.9276	0.8478	0.8510	0.8845	0.6459
(3)		√			Papillary & Follicular	0.9542	0.8279	0.8110	0.8300	0.6736
					Papillary & Medullary	0.9459	0.8324	0.8345	0.8456	0.7145
					Tertiary & Community	0.9483	0.8235	0.8214	0.8378	0.6591
(4)			√		Papillary & Follicular	0.9532	0.8245	0.8246	0.8323	0.6523
					Papillary & Medullary	0.9422	0.8334	0.8169	0.8223	0.7088
					Tertiary & Community	0.9428	0.8402	0.8449	0.8523	0.6827
(5)	√	√			Papillary & Follicular	0.8980	0.8252	0.8412	0.8475	0.7329
					Papillary & Medullary	0.9116	0.8230	0.8381	0.8491	0.7440
					Tertiary & Community	0.9324	0.8308	0.8389	0.8523	0.7157
(6)	√		√		Papillary & Follicular	0.9377	0.8414	0.8426	0.8620	0.7003
					Papillary & Medullary	0.9712	0.8091	0.8220	0.8432	0.7145
					Tertiary & Community	0.9739	0.8130	0.8249	0.8414	0.7289
(7)	√	√	√		Papillary & Follicular	0.9382	0.8208	0.8422	0.8621	0.7575
					Papillary & Medullary	0.9005	0.8048	0.8112	0.8374	0.7329
					Tertiary & Community	0.8892	0.8331	0.8213	0.8375	0.7451
(8)	√	√		√	Papillary & Follicular	0.9239	0.8312	0.8423	0.8528	0.7555
					Papillary & Medullary	0.9291	0.8002	0.8199	0.8256	0.7679
					Tertiary & Community	0.9210	0.8293	0.8377	0.8420	0.7761
**(9)**	√	√	√	√	**Papillary & Follicular**	**0.9768**	**0.8784**	**0.8672**	**0.8893**	**0.8098**
					**Papillary & Medullary**	**0.9382**	**0.8621**	**0.8572**	**0.8688**	**0.7832**
					**Tertiary & Community**	**0.9091**	**0.8621**	**0.8693**	**0.8772**	**0.7794**

Supplementary Table 1: Ablation study for components of the proposed QP-Net. a. (1) ~ (9): The empirical results support that each component contributes to the overall model performance. b. (2) vs. (5): the adversarial structure contributes to model performance on less-prevalent subgroups. c. (2) vs. (7): empirical evidence on the effectiveness of marginal domain invariance on our thyroid ultrasound dataset. d. (5) vs. (6) vs. (7) and (8) vs. (9): combining adversarial structure and MMD loss yields better performance. e. (3) vs. (5), and (4) vs. (6): multi-task learning module contributes to preserving model performance on dominant subgroup. f. (5) vs. (8), and (7) vs. (9): BSS loss contributes to the improvement of model performance on less-prevalent subgroup. Each set of experiments was repeated 20 times and the mean value of the results was recorded. Source data are provided as a Source Data file.

Type	Methods			Subgroup (Dominant & Less-prevalent)	AUROC				
	Name	Data load	Training Process		Train	Valid	Test	Test (Dominant)	Test (Less-prevalent)
Simple Group Balancing	I. Balanced−ERM[1]	Upsampling minority groups	ERM	Papillary & Follicular	0.9232	0.7412	0.8672	0.8710	0.7423
				Papillary & Medullary	0.9471	0.8212	0.8512	0.8527	0.7622
				Tertiary & Community	0.9277	0.7923	0.8591	0.8680	0.7283
	Ours	Upsampling minority groups	Quasi Pareto	Papillary & Follicular	0.9819	0.8511	0.8631	0.8721	0.7988
				Papillary & Medullary	0.9125	0.8518	0.8512	0.8621	0.7842
				Tertiary & Community	0.9013	0.8710	0.8628	0.8784	0.7512
	II. SUBG[2]	Subsampling majority groups	ERM	Papillary & Follicular	0.9459	0.8343	0.8213	0.8452	0.6709
				Papillary & Medullary	0.9672	0.8500	0.8490	0.8544	0.6862
				Tertiary & Community	0.9362	0.8322	0.8352	0.8465	0.7060
	Ours	Subsampling majority groups	Quasi Pareto	Papillary & Follicular	0.9102	0.8523	0.8484	0.8546	0.7772
				Papillary & Medullary	0.9491	0.8600	0.8520	0.8527	0.7592
				Tertiary & Community	0.9441	0.8662	0.8677	0.8755	0.7101
Minimax Approach	III. Minimax Pareto fairness[3]	Common	Minimax Pareto Fair Optimization	Papillary & Follicular	0.9667	0.8392	0.8012	0.8256	0.7931
				Papillary & Medullary	0.9392	0.8101	0.8173	0.8378	0.7693
				Tertiary & Community	0.9102	0.7972	0.8087	0.8182	0.7771
Domain Invariance	IV. Domain-Adversarial NeuralNetwork[4]	Common	ERM + Maximize domain classifier loss	Papillary & Follicular	0.8906	0.8407	0.8381	0.8493	0.7438
				Papillary & Medullary	0.9321	0.8205	0.8304	0.8349	0.6779
				Tertiary & Community	0.8964	0.8417	0.8333	0.8436	0.7417
Dynamic Weighted Loss	V. Dynamically Weighted Balanced Loss[5]	Common	ERM + DWS Loss	Papillary & Follicular	0.9432	0.8326	0.8438	0.8624	0.7533
				Papillary & Medullary	0.9121	0.8534	0.8223	0.8552	0.7563
				Tertiary & Community	0.9372	0.8171	0.8241	0.8439	0.7474
Optimized loss functions	VI. Focal Loss[6]	Common	ERM + Focal Loss	Papillary & Follicular	0.9485	0.8578	0.8532	0.8669	0.7200
				Papillary & Medullary	0.9651	0.8313	0.8309	0.8474	0.6659
				Tertiary & Community	0.9712	0.8434	0.8455	0.8619	0.7111
Ours	Quasi Pareto	Common	$L_y(\omega_Z, \omega_y)$ $-L_{\text{Class conditional}}$[7] $+\gamma_{MMD}L_{MMD}(\omega_Z)$ $+\gamma_{BSS}L_{BSS}(\omega_Z)$	Papillary & Follicular	0.9504	0.8231	0.8425	0.8531	0.7021
				Papillary & Medullary	0.9231	0.8191	0.8302	0.8462	0.7482
				Tertiary & Community	0.9011	0.8245	0.8110	0.8366	0.7531
		Common	$\boldsymbol{L_y(\omega_Z, \omega_y)}$ $\boldsymbol{-\gamma_d L_d(\omega_Z, \omega_y)}$[7] $\boldsymbol{+\gamma_{MMD}L_{MMD}(\omega_Z)}$ $\boldsymbol{+\gamma_{BSS}L_{BSS}(\omega_Z)}$	**Papillary & Follicular**	**0.9768**	**0.8784**	**0.8672**	**0.8893**	**0.8098**
				**Papillary & Medullary**	**0.9382**	**0.8621**	**0.8572**	**0.8688**	**0.7832**
				**Tertiary & Community**	**0.9091**	**0.8621**	**0.8693**	**0.8772**	**0.7794**

Supplementary Table 2: Method comparison with state-of-art fairness and domain adaptation approaches. The lowest row (highlighted in gray) represents the standard settings of the proposed QP-Net. Rows 2, 4 (with the Name 'Ours') also utilize QP-Net for the training process but incorporate additional data load strategy compared to the standard settings. The results support that existing methods cannot achieve fairness in both dominant and less-prevalent subgroups without both components (multi-task learning and domain adaptation) of the proposed QPI approach. Specifically, the results in rows 2 and 4 (with the Name 'Ours') suggest that subgroup sampling strategies do not yield additional performance improvements for the QP-Net. Each set of experiments was repeated 20 times and the mean value of the results was recorded. Source data are provided as a Source Data file.

	Hyperparameter comparison (γ)		Subgroup (Dominant & Less-prevalent)	AUROC				
	γ_d	γ_{MMD}		Train	Valid	Test	Test (Dominant)	Test (Less-prevalent)
(1)	0	0	Papillary & Follicular	0.8980	0.8252	0.8412	0.8475	0.7329
			Papillary & Medullary	0.9116	0.8430	0.8381	0.8491	0.7440
			Tertiary & Community	0.9324	0.8508	0.8389	0.8523	0.7157
(2)	0.1	0	Papillary & Follicular	0.9790	0.8466	0.8460	0.8617	0.7189
			Papillary & Medullary	0.9871	0.8505	0.8524	0.8688	0.7236
			Tertiary & Community	0.9909	0.8413	0.8495	0.8665	0.6950
(3)	0.2	0	Papillary & Follicular	0.9887	0.8278	0.8416	0.8597	0.6967
			Papillary & Medullary	0.9732	0.8305	0.8381	0.8502	0.7472
			Tertiary & Community	0.9391	0.8179	0.8080	0.8243	0.6813
(4)	0.3	0	Papillary & Follicular	0.9732	0.8305	0.8381	0.8502	0.7472
			Papillary & Medullary	0.9472	0.8360	0.8288	0.8378	0.7569
			Tertiary & Community	0.9759	0.8354	0.8329	0.8453	0.7419
(5)	0.4	0	Papillary & Follicular	0.9194	0.8247	0.8334	0.8512	0.7086
			Papillary & Medullary	0.9607	0.8436	0.8416	0.8604	0.6823
			Tertiary & Community	0.9825	0.8454	0.8496	0.8713	0.6756
(6)	0	1	Papillary & Follicular	0.9792	0.8403	0.8429	0.8586	0.7111
			Papillary & Medullary	0.9806	0.7755	0.8423	0.8618	0.7017
			Tertiary & Community	0.9812	0.8482	0.8466	0.8624	0.6827
(7)	0.1	1	Papillary & Follicular	0.9079	0.8641	0.8519	0.8800	0.7772
			Papillary & Medullary	0.9069	0.8403	0.8552	0.8762	0.7557
			Tertiary & Community	0.9479	0.8598	0.8527	0.8686	0.7296
(8)	**0.2**	**1**	Papillary & Follicular	**0.9768**	**0.8784**	**0.8672**	**0.8893**	**0.8098**
			Papillary & Medullary	**0.9382**	**0.8621**	**0.8572**	**0.8688**	**0.7832**
			Tertiary & Community	**0.9091**	**0.8621**	**0.8693**	**0.8772**	**0.7687**
(9)	0.3	1	Papillary & Follicular	0.9631	0.8377	0.8509	0.8641	0.7641
			Papillary & Medullary	0.9079	0.8341	0.8319	0.8435	0.7559
			Tertiary & Community	0.9069	0.8303	0.8325	0.8464	0.7157
(10)	0.4	1	Papillary & Follicular	0.9938	0.8060	0.8139	0.8514	0.7506
			Papillary & Medullary	0.9634	0.8341	0.8382	0.8562	0.7490
			Tertiary & Community	0.9321	0.8352	0.8499	0.8623	0.7578
(11)	0	2	Papillary & Follicular	0.9948	0.8162	0.8023	0.8512	0.6453
			Papillary & Medullary	0.9859	0.8023	0.8376	0.8500	0.6479
			Tertiary & Community	0.9050	0.8299	0.8150	0.8516	0.6500
(12)	0.1	2	Papillary & Follicular	0.8907	0.8247	0.8252	0.8467	0.6373
			Papillary & Medullary	0.8907	0.8241	0.8247	0.8463	0.6369
			Tertiary & Community	0.8901	0.8217	0.8241	0.8450	0.6433
(13)	0.2	2	Papillary & Follicular	0.8817	0.8313	0.8340	0.8475	0.7101
			Papillary & Medullary	0.8866	0.8308	0.8335	0.8471	0.6442
			Tertiary & Community	0.8850	0.8309	0.8338	0.8473	0.7109
(14)	0.3	2	Papillary & Follicular	0.9414	0.8299	0.8319	0.8487	0.6803
			Papillary & Medullary	0.9407	0.8324	0.8315	0.8499	0.6716
			Tertiary & Community	0.9186	0.8265	0.8264	0.8467	0.6463
(15)	0.4	2	Papillary & Follicular	0.9382	0.8353	0.8328	0.8393	0.5377
			Papillary & Medullary	0.9732	0.8055	0.8032	0.8300	0.6792
			Tertiary & Community	0.9412	0.8325	0.8380	0.8485	0.7485

Supplementary Table 3: Hyperparameter comparison results of all three pairs of subgroups on our thyroid ultrasound dataset. Hyperparameters used in the experiment are determined based on empirical results, and the optimal hyperparameter combination yielded the best model performance on all three pairs of subgroups. Each set of experiments was repeated 20 times and the mean value of the results was recorded. Source data are provided as a Source Data file.

Supplementary Figures

a

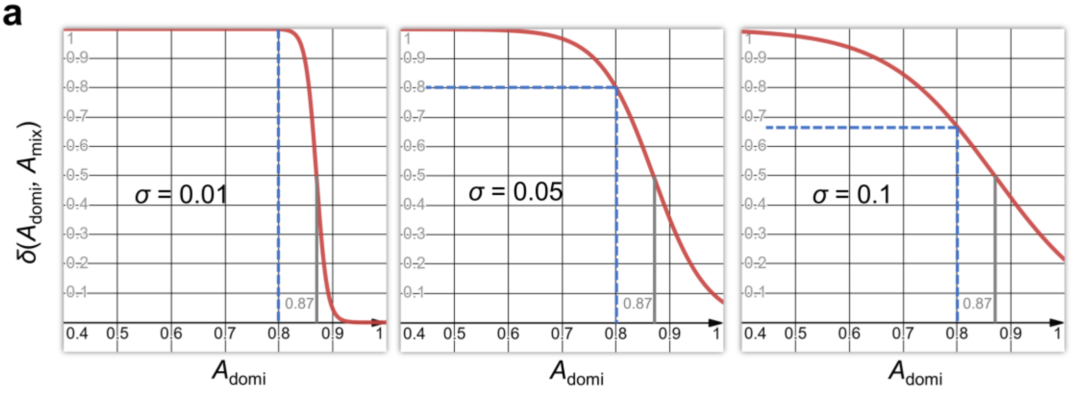

b

	Hyperparameter comparison (γ)				Subgroup (Dominant & Less-prevalent)	AUROC				
	**σ**	**γ_d**	**γ_{MMD}**	**γ_{BSS}**		**Train**	**Valid**	**Test**	**Test (Dominant)**	**Test (Less-prevalent)**
(1)	0.01	0.2	1	0.5	Papillary & Follicular	0.9423	0.8553	0.8225	0.8374	0.7623
					Papillary & Medullary	0.9422	0.8534	0.8373	0.8423	0.7540
					Tertiary & Community	0.9534	0.8463	0.8255	0.8423	0.7657
(2)	**0.05**				Papillary & Follicular	**0.9768**	**0.8784**	**0.8672**	**0.8893**	**0.8098**
					Papillary & Medullary	**0.9382**	**0.8621**	**0.8572**	**0.8688**	**0.7832**
					Tertiary & Community	**0.9091**	**0.8621**	**0.8693**	**0.8772**	**0.7794**
(3)	0.1				Papillary & Follicular	0.9342	0.8653	0.8553	0.8797	0.6367
					Papillary & Medullary	0.9530	0.8564	0.8581	0.8602	0.6772
					Tertiary & Community	0.9535	0.8554	0.8503	0.8643	0.6834
(4)	0.01			1	Papillary & Follicular	0.9727	0.8432	0.8400	0.8450	0.7232
					Papillary & Medullary	0.9827	0.8676	0.8423	0.8634	0.7540
					Tertiary & Community	0.9762	0.8611	0.8354	0.8644	0.7435
(5)	0.5				Papillary & Follicular	0.9748	0.8625	0.8643	0.8743	0.7020
					Papillary & Medullary	0.9727	0.8332	0.8145	0.8442	0.7321
					Tertiary & Community	0.9861	0.8655	0.7934	0.8223	0.6981
(6)	0.1				Papillary & Follicular	0.9846	0.7632	0.7533	0.7932	0.6428
					Papillary & Medullary	0.9625	0.7653	0.7523	0.7943	0.6782
					Tertiary & Community	0.9542	0.7540	0.7356	0.7534	0.6550

Supplementary Figure 1: Hyperparameter comparison experiments of σ. **a** σ governs the extent of minor weight adjustments, and larger σ values yield a smoother $\delta(A_{\text{domi}}, A_{\text{mix}})$ curve. **b** Empirical results demonstrate that $\sigma = 0.05$ emerges as a preferable parameter selection. Each set of experiments was repeated 20 times and the mean value of the results was recorded.

Explanation for Supplementary Figure 1: **a** Within the QPI framework, we have employed a loss function denoted as L_y with adaptive weights in the multi-task classifier. The hyperparameter σ governs the extent of minor weight adjustments. Supplementary Figure 1 provides the results of our comparison experiments on different values of σ. Specifically, in Supplementary Figure 1a, as σ increases, the curve of $\delta(A_{\text{domi}}, A_{\text{mix}})$ appears more gradual. We assume $A_{\text{mix}} = 0.87$ (dotted line), when $A_{\text{domi}} < A_{\text{mix}}$, the weight of the dominant yields a $\delta > 0.5$. We prefer $\sigma = 0.05$, which has a moderate slope and appropriate weight changes. For $A_{\text{domi}} \in [0.6, 1]$, we have $\delta \in [0, 0.99]$. This range exhibits variability in response to changes in A_{mix}. For example, as $\sigma = 0.05$, when $A_{\text{domi}} = 0.8$, we have $\delta(A_{\text{domi}}, A_{\text{mix}}) = 0.8022$, which is better than $\delta = 1$ and $\delta = 0.66$ (solid line) in our experiment. Therefore, considering all factors, $\sigma = 0.05$ emerges as a preferable parameter selection. **b** Comparative experiment for σ and γ_{BSS} parameters. Make sure that γ_{d}, γ_{MMD}

parameter is unchanged and compare the value of σ and γ_{BSS}. According to AUC, $\sigma = 0.05$ and $\gamma_{\mathrm{BSS}} = 0.5$ is the better choice and achieves better results in all three groups. Each set of experiments was repeated 20 times and the mean value of the results was recorded. Source data are provided as a Source Data file.

Supplementary Methods

Name list of hospitals for thyroid data collection

We provide the full name list of nine tertiary hospitals and one community hospital for our thyroid ultrasound image collection.

Tertiary hospitals:

Shanghai Tongren Hospital, Jiuquan Hospital of Shanghai General Hospital, Shanghai Pudong People's Hospital, Xuzhou City Central Hospital, Nanjing Drum Tower Hospital, Affiliated Dongyang Hospital of Wenzhou Medical University, Shanghai Tenth People's Hospital, Shanghai No. 4 People's Hospital, Inner Mongolia Xing'an Meng People's Hospital

Community hospital:

Yiwu Central Hospital

Experimental details for nnU-net

We employed the Attention U-Net segmentation network for image edge cropping. The input dimensions of the model were set at 256 * 256. To begin, a series of five convolutional pooling layers were utilized to extract features. The number of convolutional features in each layer was sequentially set at 64, 128, 256, 512, and 1024, thereby forming the encoding component of the network. In the decoding aspect of the architecture, four convolutional layers were incorporated, each enhanced with an attention mechanism. These layers facilitated the generation of the final output. Throughout the training process of the model, a batch size of 8 was selected, and the Adam optimizer was employed with a learning rate of 0.001. The training was conducted over a maximum of 300 epochs. 1200 PTC samples from tertiary hospitals manually cropped by specialists with over 5 years of ultrasound imaging experience were used as training and validation data, following a 4:1 ratio. The IOU achieved by the employed Attention U-Net on our comprehensive thyroid ultrasound dataset reached an impressive value of 0.97. All the instances of cropping failure were subsequently rectified through manual intervention, amounting to a correction ratio of 4.27%.

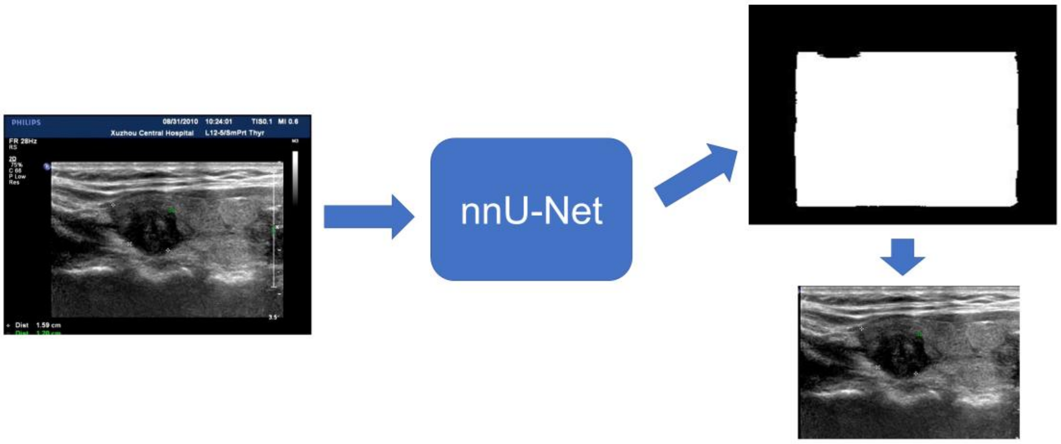

Supplementary Figure 2: Illustration of image edge cropping utilizing nnU-Net.

Training details

We set the training objectives for $\mathcal{F}_y$ and $\mathcal{F}_d$ to produce the most precise predictions possible using the features extracted by $\mathcal{F}_z$. Nevertheless, the training objectives for $\mathcal{F}_z$ are twofold: first, to minimize the error of $\mathcal{F}_y$, and second, to maximize the error of $\mathcal{F}_d$. These training objectives can simultaneously guarantee that the extracted features satisfy two properties: discrimination and domain-invariance. Consequently, we determine the optimal model weights based on the loss function using the following formula:

$$\widehat{\boldsymbol{\omega}}_z = \underset{\omega_z}{\operatorname{argmin}}[\mathcal{L}_y(\boldsymbol{\omega}_z, \boldsymbol{\omega}_y) - \gamma_d \mathcal{L}_d(\boldsymbol{\omega}_z, \boldsymbol{\omega}_d) + \gamma_{\mathrm{MMD}} \mathcal{L}_{\mathrm{MMD}}(\boldsymbol{\omega}_z) + \gamma_{\mathrm{BSS}} \mathcal{L}_{\mathrm{BSS}}(\boldsymbol{\omega}_z)] \quad (22)$$

$$\widehat{\boldsymbol{\omega}}_y = \underset{\omega_y}{\operatorname{argmin}} \mathcal{L}_y(\boldsymbol{\omega}_z, \boldsymbol{\omega}_y) \quad (23)$$

$$\widehat{\boldsymbol{\omega}}_d = \underset{\omega_d}{\operatorname{argmin}} \mathcal{L}_d(\boldsymbol{\omega}_z, \boldsymbol{\omega}_d) \quad (24)$$

We used backpropagation to update the model parameters step by step during the training process. Under the learning rate η, the parameter update formula is as follows:

$$\begin{aligned} \Delta\boldsymbol{\omega}_z &= -\eta \frac{\partial \mathcal{L}}{\partial \boldsymbol{\omega}_z} \\ &= -\eta \left(\frac{\partial \mathcal{L}_y}{\partial \boldsymbol{\omega}_z} - \gamma_d \frac{\partial \mathcal{L}_d}{\partial \boldsymbol{\omega}_z} + \gamma_{\mathrm{MMD}} \frac{\partial \mathcal{L}_{\mathrm{MMD}}}{\partial \boldsymbol{\omega}_z} + \gamma_{\mathrm{BSS}} \frac{\partial \mathcal{L}_{\mathrm{BSS}}}{\partial \boldsymbol{\omega}_z} \right) \end{aligned} \quad (25)$$

$$\begin{aligned} \Delta\boldsymbol{\omega}_y &= -\eta \frac{\partial \mathcal{L}}{\partial \boldsymbol{\omega}_y} \\ &= -\eta \frac{\partial \mathcal{L}_y}{\partial \boldsymbol{\omega}_y} \end{aligned} \quad (26)$$

$$\Delta\boldsymbol{\omega}_d = -\eta \frac{\partial \mathcal{L}_d}{\partial \boldsymbol{\omega}_d} \quad (27)$$

where η is the learning rate.

Hyperparameters are tuned on specific datasets. We attempted various combinations of hyperparameters and chose the set of hyperparameters that yielded the best model performance. Detailed hyperparameter test results are provided in Supplementary materials.

Confidence interval estimation, AUROC and Brier Score

(1) Confidence interval estimation

Suppose a normally distributed population $X \sim N(\mu, \sigma^2)$ with unknown mean μ and variance σ^2. Denote n independent samples from X as $\{X_1, X_2, \ldots, X_n\}$, and denote the sample mean as $\bar{X}$ and sample variance as S^2. Then,

$$\left[\bar{X} - t_{n-1;\frac{\alpha}{2}} \frac{S}{\sqrt{n}}, \bar{X} + t_{n-1;\frac{\alpha}{2}} \frac{S}{\sqrt{n}}\right] \tag{28}$$

is the theoretical confidence interval for μ under the confidence level of $1 - \alpha$, where $t_{n-1;\frac{\alpha}{2}}$ is the $(1 - \alpha/2)$ percentile of t-distribution with $n - 1$ degrees of freedom[8]. When $n \to +\infty$ and $\alpha = 0.5$, $t_{n-1;\frac{0.5}{2}} \to 1.96$.

In the experiment, under certain settings, we treated model prediction performance (AUROC) as a specific population and used Bootstrapping as a sampling strategy to estimate a 95% confidence interval (CI) for the mean value of the population, which in this case, is the mean prediction performance of the model (mean AUROC).

(2) AUROC calculation

The primary objective of performance evaluation in this study is to compare the performance of multiple neural network models and demonstrate superior improvement. Accordingly, the area under the ROC curve (AUROC)[9] was utilized to assess the performance of neural network models.

In binary classification tasks, a binary classifier's prediction results can be categorized as: True positive, True negative, False positive, and False negative. The criteria for their classification are shown in Supplementary Table 4. The label represents the actual situation of the sample, whereas Prediction is the classification output of the model. We use the following formulas to calculate the AUC values using these four types of classification results.

		Label	
		Benign	Malignant
Prediction	Benign	Truepositive	Falsepositive
	Malignant	Falsenegative	Truenegative

Supplementary Table 4. The prediction results of binary classifiers can be divided into the four categories.

$$\text{AUROC} = \int_0^1 \frac{\text{Truepositive}}{\text{Truepositive+Falsenegative}} \left\{ \left(\frac{\text{Falsepositive}}{\text{Truenegative+Falsepositive}} \right)^{-1} \right\} \mathrm{dx} \tag{29}$$

(3) Brier Score

The Brier Score is a measure of the difference between probability and true label results[10]. The Brier score ranges from 0 to 1, and the higher the score, the worse the prediction and the worse the calibration, so the closer the Brier score is to 0, the better.

$$\mathrm{BS} = \frac{1}{N}\sum_{t=1}^{N}(y_{\mathrm{pred}} - y_{\mathrm{label}})^2$$

Where, y_{pred} is predicted probability, y_{label} is the true label.

Reference

1. Zhang, H.*, et al.* Improving the fairness of chest x-ray classifiers. in *Conference on Health, Inference, and Learning* 204-233 (PMLR, 2022).
2. Idrissi, B.Y., Arjovsky, M., Pezeshki, M. & Lopez-Paz, D. Simple data balancing achieves competitive worst-group-accuracy. in *Conference on Causal Learning and Reasoning* 336-351 (PMLR, 2022).
3. Martinez, N., Bertran, M. & Sapiro, G. Minimax pareto fairness: A multi objective perspective. in *International Conference on Machine Learning* 6755-6764 (PMLR, 2020).
4. Ganin, Y.*, et al.* Domain-adversarial training of neural networks. *The journal of machine learning research* **17**, 2096-2030 (2016).
5. Fernando, K.R.M. & Tsokos, C.P. Dynamically weighted balanced loss: class imbalanced learning and confidence calibration of deep neural networks. *IEEE Transactions on Neural Networks and Learning Systems* **33**, 2940-2951 (2021).
6. Mukhoti, J.*, et al.* Calibrating deep neural networks using focal loss. *Advances in Neural Information Processing Systems* **33**, 15288-15299 (2020).
7. Lee, W., Kim, H. & Lee, J. Compact class-conditional domain invariant learning for multi-class domain adaptation. *Pattern Recognition* **112**, 107763 (2021).
8. Ci, B., & Rule, R. O. (1987). Confidence intervals. Lancet, 1(8531), 494-7.
9. Fawcett, Tom (2006); An introduction to ROC analysis, Pattern Recognition Letters, 27, 861–874.
10. Brier, G. W. (1950). Verification of forecasts expressed in terms of probability. Monthly weather review, 78(1), 1-3.
