# Supplementary material for: Enhancing the fairness of AI prediction models by Quasi-Pareto improvement among heterogeneous thyroid nodule population
Source: Nat Commun. 2024 Mar 4;15:1958. doi: 10.1038/s41467-024-44906-y (PMC10912763; doi:10.1038/s41467-024-44906-y)
Supplement: Supplementary file 2 — Reporting Summary [file 41467_2024_44906_MOESM2_ESM.pdf]

nature portfolio

Corresponding author(s): Hui Lu

Last updated by author(s): Nov 27, 2023

Reporting Summary

Nature Portfolio wishes to improve the reproducibility of the work that we publish. This form provides structure for consistency and transparency in reporting. For further information on Nature Portfolio policies, see our Editorial Policies and the Editorial Policy Checklist.

Statistics

For all statistical analyses, confirm that the following items are present in the figure legend, table legend, main text, or Methods section.

n/a	Confirmed	
☐	☒	The exact sample size (n) for each experimental group/condition, given as a discrete number and unit of measurement
☐	☒	A statement on whether measurements were taken from distinct samples or whether the same sample was measured repeatedly
☒	☐	The statistical test(s) used AND whether they are one- or two-sided<br>*Only common tests should be described solely by name; describe more complex techniques in the Methods section.*
☒	☐	A description of all covariates tested
☒	☐	A description of any assumptions or corrections, such as tests of normality and adjustment for multiple comparisons
☐	☒	A full description of the statistical parameters including central tendency (e.g. means) or other basic estimates (e.g. regression coefficient) AND variation (e.g. standard deviation) or associated estimates of uncertainty (e.g. confidence intervals)
☒	☐	For null hypothesis testing, the test statistic (e.g. F, t, r) with confidence intervals, effect sizes, degrees of freedom and P value noted<br>*Give P values as exact values whenever suitable.*
☒	☐	For Bayesian analysis, information on the choice of priors and Markov chain Monte Carlo settings
☒	☐	For hierarchical and complex designs, identification of the appropriate level for tests and full reporting of outcomes
☒	☐	Estimates of effect sizes (e.g. Cohen's d, Pearson's r), indicating how they were calculated

Our web collection on statistics for biologists contains articles on many of the points above.

Software and code

Policy information about availability of computer code

Data collection	The ultrasound image data is collected from the hospitals and downloaded independently from the hospital information systems by the doctors in PNG format with different sizes. The authors had access to anonymized data only. The images are cut and scaled down to 512x512 pixels to ensure that the internal information of the pictures is not lost and not deformed. The ultrasound and pathology reports are in PDF format. No software was used for data collection.
Data analysis	The experiments were carried out with python (version 3.8) and mase use of the following packages: torch (version 1.10.0+cu111), torchvision (0.11.0+cu111), numpy (version 1.19.5); scikit-learn (version 1.0.2), pandas (version 1.3.5), openslide-python (1.1.2), matplotlib (version 3.5.1), scipy (version 1.8.0); opencv-python(version 4.5.5.62); Pillow(version 8.4.0);torch-cka(version 0.21)<br>QP-Net are available in https://github.com/fangdai-dear/QuasiParetoimprovement<br>Segmentation model are available in https://github.com/MIC-DKFZ/nnUNet.<br>CKA code is adapted from https://github.com/AntixK/PyTorch-Model-Compare.git, the adapted version is in https://github.com/fangdai-dear/QuasiParetoimprovement.

For manuscripts utilizing custom algorithms or software that are central to the research but not yet described in published literature, software must be made available to editors and reviewers. We strongly encourage code deposition in a community repository (e.g. GitHub). See the Nature Portfolio guidelines for submitting code & software for further information.

Data

Policy information about availability of data

All manuscripts must include a data availability statement. This statement should provide the following information, where applicable:

- Accession codes, unique identifiers, or web links for publicly available datasets
- A description of any restrictions on data availability
- For clinical datasets or third party data, please ensure that the statement adheres to our policy

The MICCAI 2020 TN-SCUI ultrasound images of thyroid nodules used in this study are made available at https://github.com/fangdai-dear/QuasiParetoImprovement.
Partial thyroid ultrasonography data used in this study are subject to privacy restrictions, but may be anonymized and made available upon request to the corresponding authors at huilu@sjtu.edu.cn, who will provide a response within 14 days and supply the data use agreement limiting its usage to non-commercial research purposes.
The CheXpert public dataset is available at https://stanfordmlgroup.github.io/competitions/CheXpert/. The ISIC2019 Skin Image public dataset is available at https://challenge.isic-archive.com/data/#2019.

Research involving human participants, their data, or biological material

Policy information about studies with human participants or human data. See also policy information about sex, gender (identity/presentation), and sexual orientation and race, ethnicity and racism.

Reporting on sex and gender	Gender-related experiments and results are reported in manuscript
Reporting on race, ethnicity, or other socially relevant groupings	None
Population characteristics	The study included 360,455 thyroid ultrasound images from 123,301 patients over the course of nearly a decade at nine top-tier hospitals and one community hospital in China . All ultrasound data were annotated by specialists with more than five years of experience and included comprehensive ultrasound reports and aspiration biopsy pathology reports. For histological subtypes, PTC accounted for 87%, FTC for 8.7%, and MTC for 4.2%; for age, patient age ranged from 0 to 85 years, with the 35–60 age group being the largest at 48%; for gender, the male-to-female ratio was 0.49; for nodule sizes, they ranged from 0.3cm to 11.3cm, with the <1cm group accounting for 58%. For hospitals, the tertiary-to-community ratio was 4.2.
Recruitment	n/a
Ethics oversight	This study was approved by the institutional review board (IRB) of Shanghai Tong Ren Hospital and undertaken according to the Declaration of Helsinki. Informed consent from patients with thyroid cancer and controls was exempted by the IRB because of the retrospective nature of this study.

Note that full information on the approval of the study protocol must also be provided in the manuscript.

Field-specific reporting

Please select the one below that is the best fit for your research. If you are not sure, read the appropriate sections before making your selection.

☒ Life sciences ☐ Behavioural & social sciences ☐ Ecological, evolutionary & environmental sciences

For a reference copy of the document with all sections, see nature.com/documents/nr-reporting-summary-flat.pdf

Life sciences study design

All studies must disclose on these points even when the disclosure is negative.

Sample size	For all cohorts, sample sizes were determined based on the maximum number of samples available which respect the inclusion criteria detailed below. Largest possible sets were used from all datasets.
Data exclusions	We gathered a 10-year dataset of thyroid ultrasound images (from January 2013 to January 2023) and conducted a retrospective study at nine top-tier hospitals and one community hospital in China. Each image is evaluated by a physician with more than five years of thyroid ultrasound experience. The authors had access to anonymized data only. We initially screened all patients with thyroid nodule in electronic medical record. 148,289 patients who met one of the following criteria were included: (1) Had thyroid cancer or benign diagnosis confirmed by pathology after surgery; (2) had benign diagnosis confirmed by at least one-year follow-up from experienced radiologists. After inclusion, patients who met one of the following criteria were excluded: (1) comorbidity of other life- threatening condition; (2) lack of or incomplete preoperative ultrasound report; (3) poor ultrasound image quality; (4) controversial pathological diagnosis; (5) history of thyroid ectomy or other head-and-neck cancers. After exclusion, 123,301 patients were identified as study participants.
Replication	The experiments have been run over 500 bootstrap samples and no significant differences have been found between individual runs. Confidence intervals are provided for all results.
Randomization	For the thyroid ultrasound images, the data splits were 8:1:1. Random sampling was used to create reference and alignment sets, with 500

	repetitions to calculate confidence intervals.
Blinding	In this study, we conducted an algorithmic performance analysis using only quantitative and objective statistical measures and not qualitative evaluation was used. There was no risk of bias in the performance comparison and hence no blinding in the data analysis was considered relevant. Additionally, no human intervention or human assessment of medical images was involved

Reporting for specific materials, systems and methods

We require information from authors about some types of materials, experimental systems and methods used in many studies. Here, indicate whether each material, system or method listed is relevant to your study. If you are not sure if a list item applies to your research, read the appropriate section before selecting a response.

Materials & experimental systems

n/a	Involved in the study
☒	☐ Antibodies
☒	☐ Eukaryotic cell lines
☒	☐ Palaeontology and archaeology
☒	☐ Animals and other organisms
☒	☐ Clinical data
☒	☐ Dual use research of concern
☒	☐ Plants

Methods

n/a	Involved in the study
☒	☐ ChIP-seq
☒	☐ Flow cytometry
☒	☐ MRI-based neuroimaging

Plants

Seed stocks	*Report on the source of all seed stocks or other plant material used. If applicable, state the seed stock centre and catalogue number. If plant specimens were collected from the field, describe the collection location, date and sampling procedures.*
Novel plant genotypes	*Describe the methods by which all novel plant genotypes were produced. This includes those generated by transgenic approaches, gene editing, chemical/radiation-based mutagenesis and hybridization. For transgenic lines, describe the transformation method, the number of independent lines analyzed and the generation upon which experiments were performed. For gene-edited lines, describe the editor used, the endogenous sequence targeted for editing, the targeting guide RNA sequence (if applicable) and how the editor was applied.*
Authentication	*Describe any authentication procedures for each seed stock used or novel genotype generated. Describe any experiments used to assess the effect of a mutation and, where applicable, how potential secondary effects (e.g. second site T-DNA insertions, mosiacism, off-target gene editing) were examined.*
